# Getting crystals your crystallographer will treasure: a beginner’s guide

**DOI:** 10.1107/S2056989025007807

**Published:** 2025-09-09

**Authors:** Richard J. Staples

**Affiliations:** a Chemistry Michigan State University 578 S. Shaw Lane East Lansing MI48824 USA; Harvard University, USA

**Keywords:** crystallization, crystal growth, small molecule

## Abstract

Single crystal growth for the use of X-ray diffraction can be a major stumbling path to getting good X-ray diffraction data and results for this highly used and, in some cases, required analytical technique. The growing of good single crystals is often learned by experience and has been considered an art form for many years. Presented in this paper are some of the fundamental aspects that can help a chemist generate good single crystals to take to the crystallographer or the instrument.

## Abstract 2

Review of crystal growth properties and techniques for generating a crystal for single crystal X-ray structure analysis.

## Introduction

Over the course of my career, I have helped chemists with crystal growth of material to be analyzed by X-ray diffraction. It was mostly intensified when I moved to Harvard University and ran structures for the organic division of the department. The first version of this paper was taken, in part, from my lecture given at MIT in 1998 *Getting Crystals Your Crystallographer Will Treasure* (Staples, 1998 ▸). Over the years I have expanded and enhanced this discussion and was asked to provide this information to this new section of *Acta Crystallographica* Section E.

The interactions I have had over the years have formed the basis of this document, and I will provide some examples taken from the lecture given on crystal-growth techniques. Most of these are not new or novel and have been around for quite some time (Holden & Singer, 1960 ▸; Hulliger, 1994 ▸; Jones, 1981 ▸; Sluis *et al.*, 1989 ▸; Spingler *et al.*, 2012 ▸ Xiao *et al.*, 2024 ▸: Sommer, 2024 ▸), and most single crystal X-ray diffraction facilities have a crystal-growth section on their website.

This document treats the reader as if they are a novice at crystal growth. It is recognized that even experienced crystal growers have found some of the information in here handy to have and reference. Industrial organic crystal growth and concerns are significantly different from those of single crystals, and I refer those readers to the book by Paul *et al.* (2023 ▸).

## What is a crystal structure?

### The definition of a crystal structure

A crystal structure determines the connectivity of the atoms in a compound and the way the ions and molecules pack in three dimensions to form a solid crystalline material. In this document we refer to a *lattice* as the total assembly of all space within the crystal, which provides the structure and describes the atom arrangements. Although formally this is an abstract set of geometrical points that we place on the crystal (Nespolo, 2019 ▸), it is useful in describing the overall physical and positional aspects of the atoms in the crystal.

### What information do we get?

A crystal structure provides positive identification of a single crystal taken from a hopefully pure batch of material. This provides absolute proof (*provided it was done properly*) that the compound or complex is the stated material. It provides the exact connectivity of the atoms along with the bond distances and angles between these atoms in the solid state. This information results in the complete identification of the compound. It also provides a look at any inter- and intra­mol­ecular interactions that are present, which may provide insight into the chemistry and properties of the compound.

### Why have crystal structures become so popular?

Crystal structures are rarely incorrect, and it is now faster to achieve results! With the advance in technology for X-ray diffractometers and the increased computing power of computers, single crystal studies are rapidly becoming more routine. The ease of new refinement programs makes the routine structures relatively quick and easy for even the non-specialized scientist to solve (Xiao *et al.*, 2024 ▸). In the vast majority of cases, the positive identification of a compound leaves no interpretation of the data that may lead to incorrect assignments of the structure. It also can answer basic questions regarding bonding within the molecule which may help explain the chemistry and properties that exist.

## What do we need to bring to the laboratory?

A single crystal is required in the determination of an X-ray structure. A single crystal consists of atoms which possess long-range three-dimensional order. These typically appear as regular polyhedral shapes with well defined boundaries. A few common examples include table salt, sugar, gems, quartz and metals. Something to note: if the crystals are present in a solvent, leave them in the solvent. It is best to bring them ‘as is’ to the crystallographer. Oftentimes solvent molecules are part of the crystalline material and if they are allowed to evaporate the crystals can crack and then not be usable for study. A list of companies that supply crystallographic supplies and services can be found at https://www.iucr.org/resources/other-directories/suppliers.

Single crystal X-ray diffraction analysis cannot be performed on non-crystalline materials. These amorphous materials contain atoms that have only short-range order or are oriented completely randomly in the solid. A common example of an amorphous material is glass. Polycrystalline or very fine powders can be analyzed using a technique called powder X-ray diffraction, which provides different information about the structure. I refer the reader to these insightful papers on powder X-ray techniques: Kaduk (2025 ▸); Tedesco & Brunelli (2017 ▸).

### Crystal size

The ideal size of a crystal is one which occupies the entire X-ray beam of the diffractometer. Here at MSU the width of the beam is generally 0.3 mm. This means that the ideal crystal would be a sphere of about 0.25 mm in diameter. Although this is the ideal size, one can perform X-ray determination on smaller or larger (by cutting) crystals. The capabilities of this depend on the X-ray source, the arrangement of the atoms in the lattice, which atoms are present as well as the diffraction power of the crystals. Unfortunately, the diffraction power of the crystals will be relatively unknown until the crystals are on the diffractometer.

The shapes of crystals depend on both the internal symmetry of the material and on the relative growth rate of each of the faces. In general, the faces of the crystal that grow most rapidly are those to which the crystallizing particles are bound most securely (Perkins, 2022 ▸). These rapidly growing faces are usually the smaller, less well-developed faces. Thus, the larger faces are usually associated with directions in the crystal where there are only weak intermolecular interactions.

## Where to start? The concept of crystal growing

### Properties of the compound

Solubility is the single largest and most exploited property used to grow a single crystal. Generally, one knows a fair amount of this from the synthesis and other aspects of working with the compound. The stability and reactivity of the compound also need to be considered. One does not want to cause a reaction with the compound of interest in the solvent system that is being used to grow the crystals.

**Figure 1:**
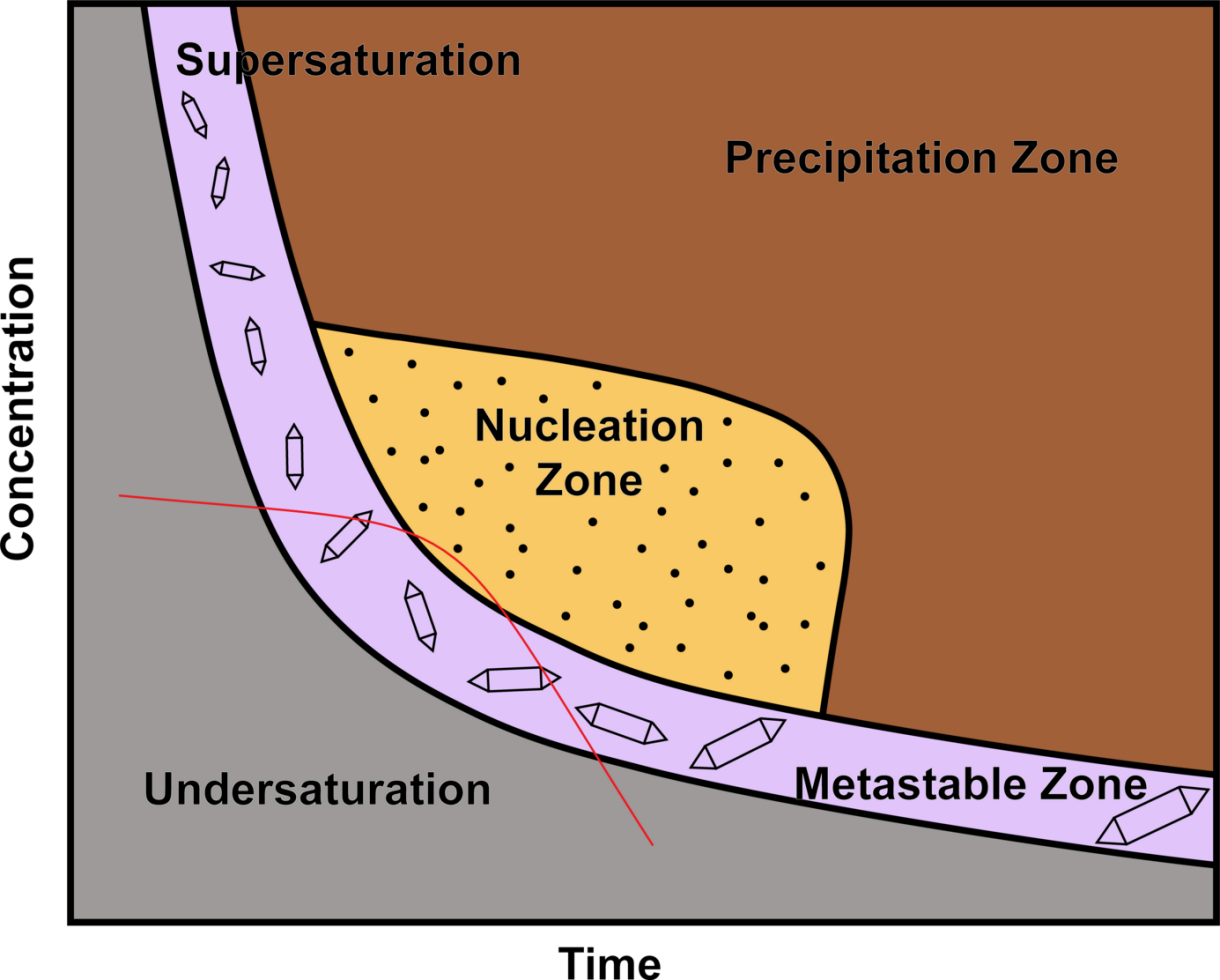
Phase diagram of crystal growth, where the red line is what we hope our solution might follow, where it spends more time in the metastable zone (crystal growth phase) than in the nucleation zone.

Nucleation (Erdemir *et al.*, 2009 ▸) and crystal growth are two separate and important steps in getting good crystals and growing large enough crystals (Fig. 1 ▸) (Xiao *et al.* 2024 ▸). Protein chemists will tell you that once the nucleation occurs, one hopes that the compound will enter the crystal-growth phase (metastable zone) in the solution (Govada & Chayen, 2019 ▸). The longer a solution remains in the nucleation zone, the smaller, more microcrystals will be obtained (large number of crystals), but if the crystal stays in the crystal-growth zone longer, then larger crystals you will be obtained (small number of crystals).

When it comes to small molecules, simple recrystallization is usually the first step in growing a good crystal. It is very important that the sample be pure and can be a solid! If the result of the experiment is an oil, this could mean that the sample is not pure since contaminants often lower the melting points of solids and can cause them to be oils. So, the first thing to do is look at the recrystallization that was performed to make the solid the first time. Was a solid formed and are these crystals well defined and good enough for X-ray study?

If these are not good enough crystals, then the next method to choose depends greatly on the physical and chemical properties of the sample. Solution methods require solubility of the solute in various solvent systems. Thermal, chemical, and melting properties can also play a major role in choosing a method for crystal growing.

***Patience is the major thing.***

For instance, I have known crystals to grow in the first attempt, while others take 10–20 trials. I’ve also worked with a few where crystal-growing experiments were unsuccessful. Sometimes crystals grow in an hour, and sometimes it takes months for them to form.

### How much material is needed?

The simple rule is that only one single crystal is needed. A good place to start is to prepare the solutions at concentration near what you would expect in order to run a typical ^1^H NMR experiment. The most important issue to consider is that the compound must be insoluble in the final resultant mixture of solvents. A quick way to estimate the amount of compound needed in a crystal can be calculated following these steps:

1. If the ideal crystal needed for X-ray diffraction is to be 0.3 × 0.3 × 0.3 mm, volume = 0.027 mm^3^.

2. The typical unit cell is 12 × 12 × 12 Å; volume = 1728 Å^3^.

3. Note that: 1 Å = 10^−10^ meters = 10^−8^ cm = 100 pm (picometres).

4. Therefore, in a typical crystal there will be 1.6 × 10^16^ unit cells.

5. If each unit cell contains eight molecules, there will be 1.3 × 10^17^ molecules in the crystal.

6. If the average molecular mass of an organic compound is = 206.2 g mol^−1^, there will be only 2.49 × 10^−7^ moles in the crystal, which is 5.1 × 10^−5^ g.

7. Take home: only about **0.051 mg** of the compound is needed for analysis by X-ray diffraction.

Unfortunately, more than one crystal grows in the vessel, so more material is needed for each experiment. Note: modern instruments and synchrotron sources are capable of analyzing crystals that are 0.1 × 0.1 × 0.1 mm or smaller.

A common starting point is to use a concentration that you would use in a typical ^1^H NMR experiment.

### What do I grow the crystals in and where?

**Figure 2:**
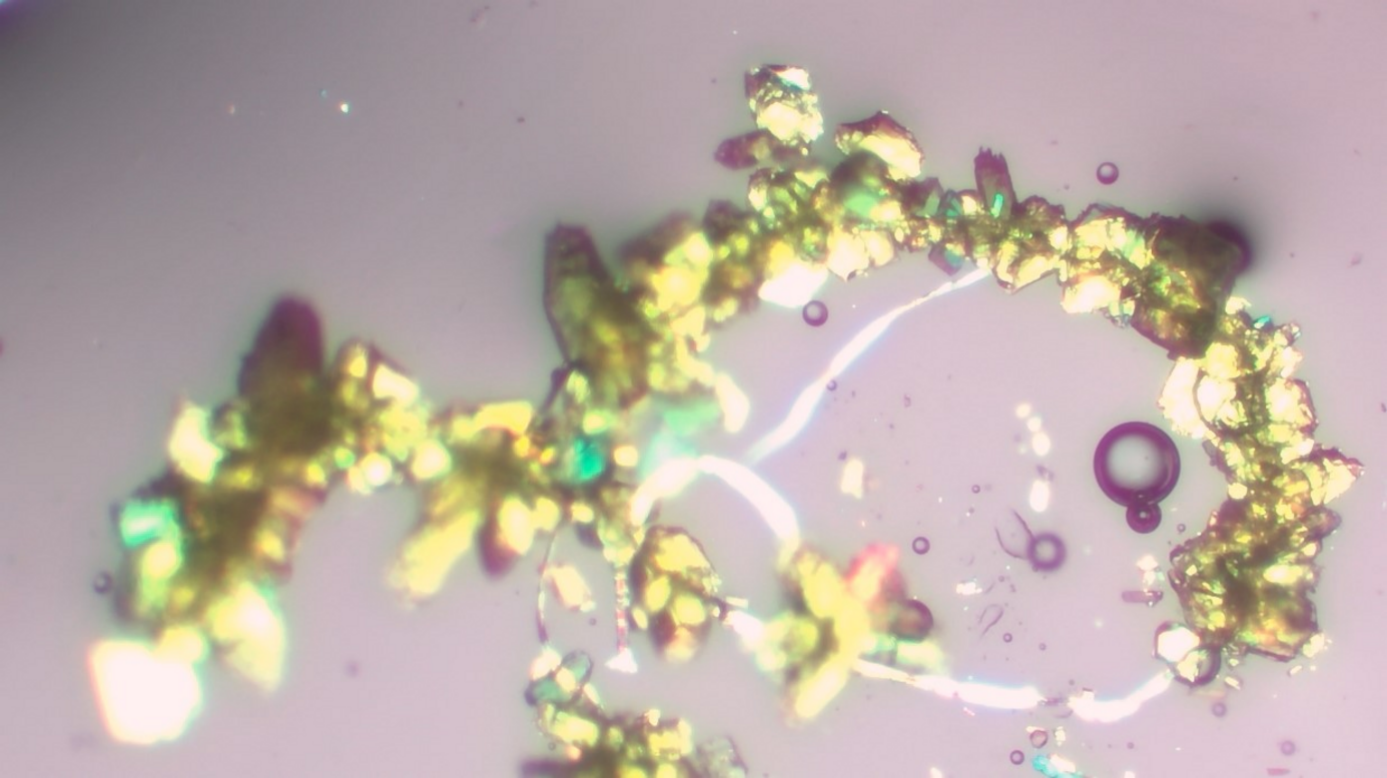
Crystals that have grown on a piece of dust particle, in this case only crystals along the dust particle were observed.

**Figure 3:**
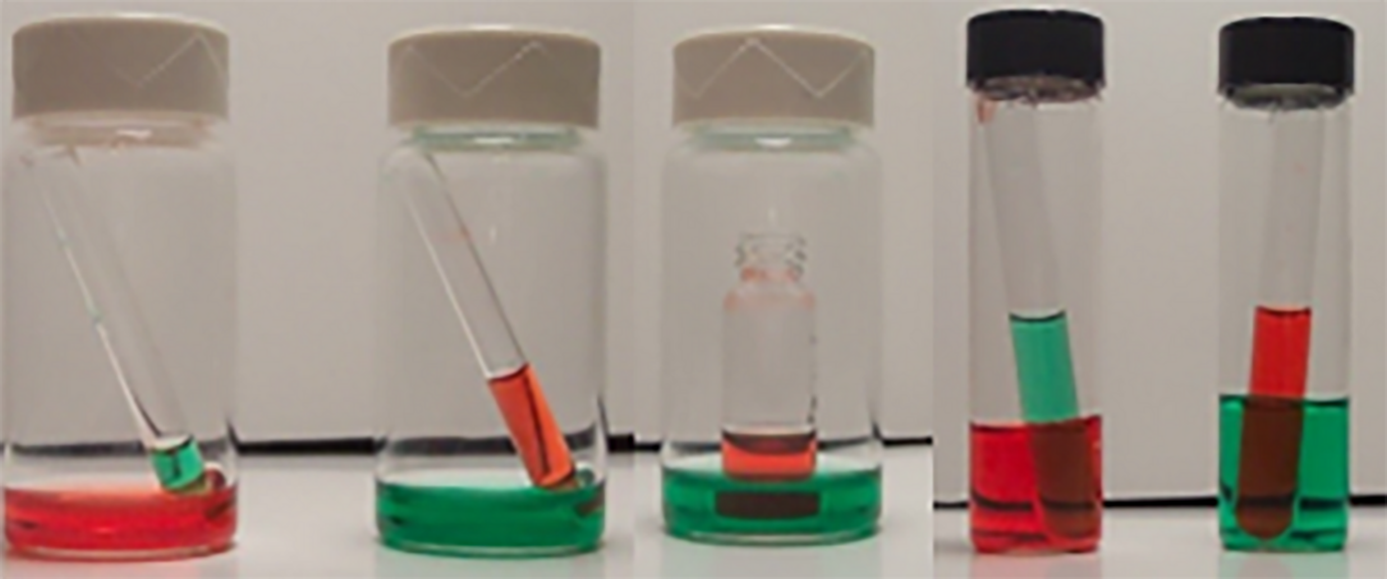
Examples of setting up vapor diffusion and possible vials one can use.

Clean glassware is very important. The use of new glassware sometimes results in problems due to the lack of nucleation sites (see Fig. 1 ▸ and §5), but this can also be helpful. Some crystallographers suggest that the new glassware contains a ‘variety of dusty contaminants’ from the manufacturing process, which may contribute to crystal growth (Fig. 2 ▸). I typically use glass vials as-are that are new from the manufacturer. Occasionally these will be cleaned and dried before use, depending on the circumstances and the chemist’s preference. I also use vials that fit inside one another (see Fig. 3 ▸).

Consider the location of the set-up. Ideally the set-up should be located out of the way where vibrations and other disturbances can be avoided. Set things up so that crystal growth can be monitored without having to move the apparatus. Since temperature can influence crystal growth, be sure to note if the apparatus is near a heater or cooler, or if it is in the sun. All these factors can change the way crystals are formed.

Keep the container covered so that no dust, dirt or spiders can enter and cause crystallization.

The use of vials that fit inside each other allows for the three most common experiments to be tried. The center vial, where the solute of interest is dissolved, consists of either a glass tube or small flat-bottom vial. Round tubes have an advantage in that they keep the material concentrated longer, which can facilitate crystallization. These work well for complexes that tend to be round or ball shaped, and it is what I usually try first here. Flat vials work better for more flat materials, and sometimes for compounds that form needles.

The outer vial is such that the cap can be tightened or left loose (slow evaporation). This also limits dust, and dirt from entering the system. The cap should be resistant to solvents.

Unfortunately, the choice of vial does not follow the above general guidelines. So, if you have trouble with one system, try the other; exceptions have been noted, in my experience.

### Solvent choice

Consider your solvents carefully. Table 1 ▸ shows some of the common solvents used to crystallize organic compounds. When designing experiments, remember that like dissolves like. If the compound is polar, then dissolve it in a polar solvent and layer that solution with non-polar solvents. Be aware that solvents such as benzene and methyl­ene chloride are either banned or considered to be controlled substances and should be used with proper safety procedures.

Avoid solvents in which your compound forms supersaturated solutions since these solutions tend to spend too much time in the nucleation phase of crystal growth. The result is crystals that are too small in size (microcrystals).

For compounds soluble in non-polar solvents, slow evaporation may be the best place to start. You could also layer with a polar solvent, but in my experience it is harder to accomplish crystal growth with this set-up.

Also remember that hydrogen bonding is very important in the crystallization process. Hydrogen bonding generally leads to more effective packing, but not always. Consider whether a hydrogen-bonding solvent might help or hinder the crystallization. Amides generally do better with hydrogen-bonding solvents.

It is amazing that some solvents tend to direct crystal growth better than other solvents. Benzene and toluene are such solvents. We have had lots of luck using some benzene (or toluene) in the solvent mixture to generate X-ray-quality crystals. The aromatic rings fill holes that may form in the lattices, but most of the time, we do not see the benzene (or toluene) co-crystallized with the compound. For many organic compounds, ethyl acetate works well.

In a perfect situation, it is best to avoid highly volatile solvents such as CH_2_Cl_2_ and di­ethyl ether since they evaporate so quickly and can result in cracked crystals. Unfortunately, these often work very well and lead to creation of crystals by slow evaporation. In this case, as stated above, be sure to bring the crystals to the crystallographer while they are in the solvent.

Other solvents to avoid are those with long alkyl chains as these often cause disorder if solvent is trapped in the lattice, since there are many conformations allowed and therefore all atoms are not in the same place throughout the lattice.

## Crystal growth

Producing good-quality crystals of a suitable size is the first and most important step in determining any crystal structure. Crystallization is the process of arranging atoms or molecules that are in a fluid or solution state into an *ordered* solid state (Laudise, 1970 ▸). This process occurs in two steps, nucleation and growth. Nucleation may occur at a seed crystal, but in the absence of seed crystals this usually occurs at some particle of dust or at some imperfection in the surrounding vessel. Crystals grow by the *ordered* deposition of material from the fluid or solution state to the surface of the crystal. For more information on crystal growth, see: *Crystal Growth of Organic Materials* (Myerson *et al.* 1996) ▸.

The main focus for growing crystals is to create an environment that changes slowly over time. This change should produce an environment in which the compound becomes supersaturated and eventually grows a solid, crystal material. This change in environment is most generally accomplished (with small molecules) by the addition of a second solvent in which the compound of interest does not dissolve.

Changing the nucleation process is the largest thing one can do, as one avoids dust or glass fragments (from pipette) being the nucleation site. If using new glass and getting lots of small crystals, scratch the glass to create only a few sites so the crystal might grow larger (see Fig. 1 ▸, §4.1).

If a sample only yields small crystals, the method should generally be altered so as to slow down the growth step. Slowing the crystal growth sometimes requires changing the method used to grow the crystals, or lowering the temperature at which the crystals are grown by placing the apparatus in a refrigerator or freezer. Please note that the concentration of the solution can have a marked effect on the results. If the solution is too concentrated, the sample will most likely stay in the nucleation zone for too long and will generate many microcrystals. This will result in crystals that are either too small or that grow on top of each other. This generally results in a cluster of tiny crystals all stuck to each other rather than one big single crystal.

Physical disturbance of the crystal growing vessel can result in smaller crystals being formed. Choose a location to grow the crystals where there are no vibrations from elevators, doors, roto-vaps, vacuum pumps *etc*. At Harvard we noticed that standard crystals would not grow and form as well when grown on the shelves that were near the door. You should set the crystals where you can view them without having to move them, or if you do, wait one week before checking on the crystals.

Patience! Some methods work in a few hours, and other methods require weeks or even months for success.

## Crystallization methods

The techniques chosen will largely depend on the chemical properties of the compound of interest: Is the compound air sensitive or moisture sensitive? Is it hygroscopic? Can it form hydrogen bonds, does it react with certain solvents *etc*.?

### Vapor diffusion

This is by far the best crystallization method to use for small molecules and works very well when only milligram quantities are available. This method requires volatile solvents and when done properly can generate a less desirable solvent system, which then allows for slow crystal growth. Table 2 ▸ provides some initial solvent choices commonly used in crystal-growth experiments. Vapor diffusion is the movement of one solvent vapor to another through a material (in this case air) to then generate a mixed-solvent system. The lower-vapor-pressured solvent moves to the higher-vapor-pressured solvent. Higher vapor pressure means less vapors leaving the solvent, allowing the other solvent space to reach the surface.

Vapor diffusion is carried out by first placing a filtered sample solution in a small vial. Choose a vial such that the solution will fill a quarter to half of the volume. Then, place this small vial inside a larger vial that contains a solvent in which the sample is insoluble (Figs. 3 ▸ and 4 ▸). The volume of the outer solvent will vary depending on (*a*) how much is required to crystallize the material, (*b*) how fast you want the vapor to diffuse, and (*c*) the size of the inner vial. The outer vial is then sealed. **Do not disturb the vessel.** Vapor from the solvent of the outer vial then diffuses into the solution in the inner vial, causing the compound to crystallize. The vertical surfaces of the inner vial should not touch the outer vial to keep the outer solution from rising by capillary action and filling the inner vial. Sometimes this technique is combined with slow cooling by placing the vials in a refrigerator to slow the diffusion of the solvents, giving more time for the crystals to grow. Vapor pressures are given in Table 3 ▸.

### Solvent layering

This is a relatively simple concept, but in practice can be difficult to execute. First choose two solvents, one that will dissolve the compound and a second that will not. Dissolve the compound in the soluble solvent and then layer the second solvent over top of this solution *very carefully*. The key is to choose solvents that are miscible in one another yet have enough of a difference in properties that an interface develops between the two solvents as you set it up. A third solvent may be used to create a buffer to slow the diffusion rate, which controls the rate of crystallization. I recommend using benzene or toluene at the interface. Once the apparatus is set up. **Do not disturb the vessel!** The rate of crystal growth depends on the concentration level and solubility of the compound in the resulting mixed-solvent system. The set-up can be placed in a refrigerator to slow the mixing of the solvents, which will give the crystals more time to grow.

### Slow evaporation

Evaporation is by far one of the easiest methods for crystallizing organic and organometallic small molecule compounds. The choice of solvent is important because it can greatly influence the mechanism of crystal growth, when the crystal begins to form, and because the solvent may be incorporated into the crystalline lattice. The rate of crystal growth can be slowed either by reducing the rate of evaporation of the solvent, having less open area in the vial, or by cooling the solution. It is important to keep the solution clean by covering it. A simple thing is to use a low-lint, absorbent paper tissue such as a ‘Kimwipe’ or a loose vial cap, but some slow the process by putting a rubber septum on the vial and then inserting a needle.

If this method provides an oil, it could be because the compound is impure. However, if you had a solid prior to the experiment, it is more likely that the compound is too soluble in the solvent chosen for evaporation. Set the experiment up again but use a different solvent, one where the compound is not as soluble or use a second, less-volatile solvent. Tetra­hydro­furan (THF) is known to cause oiling out of the compound, but it is also well known to grow crystals. Typically, get an oil once, avoid THF.

This method does not generally provide the best crystals, since the crystallization proceeds only when there is only a small amount of solvent left, causing the crystals to grow upon each other. Also, the crystals tend to adhere to the glass walls, which can make it more difficult to retrieve the crystals without damaging them.

The use of mixed solvents in this method can be done to help the crystallization, *i.e.* di­chloro­methane solution with some heptane. The di­chloro­methane will evaporate, causing an increase in the heptane concentration and then possibly crystallization of the compound.

**Figure 5:**
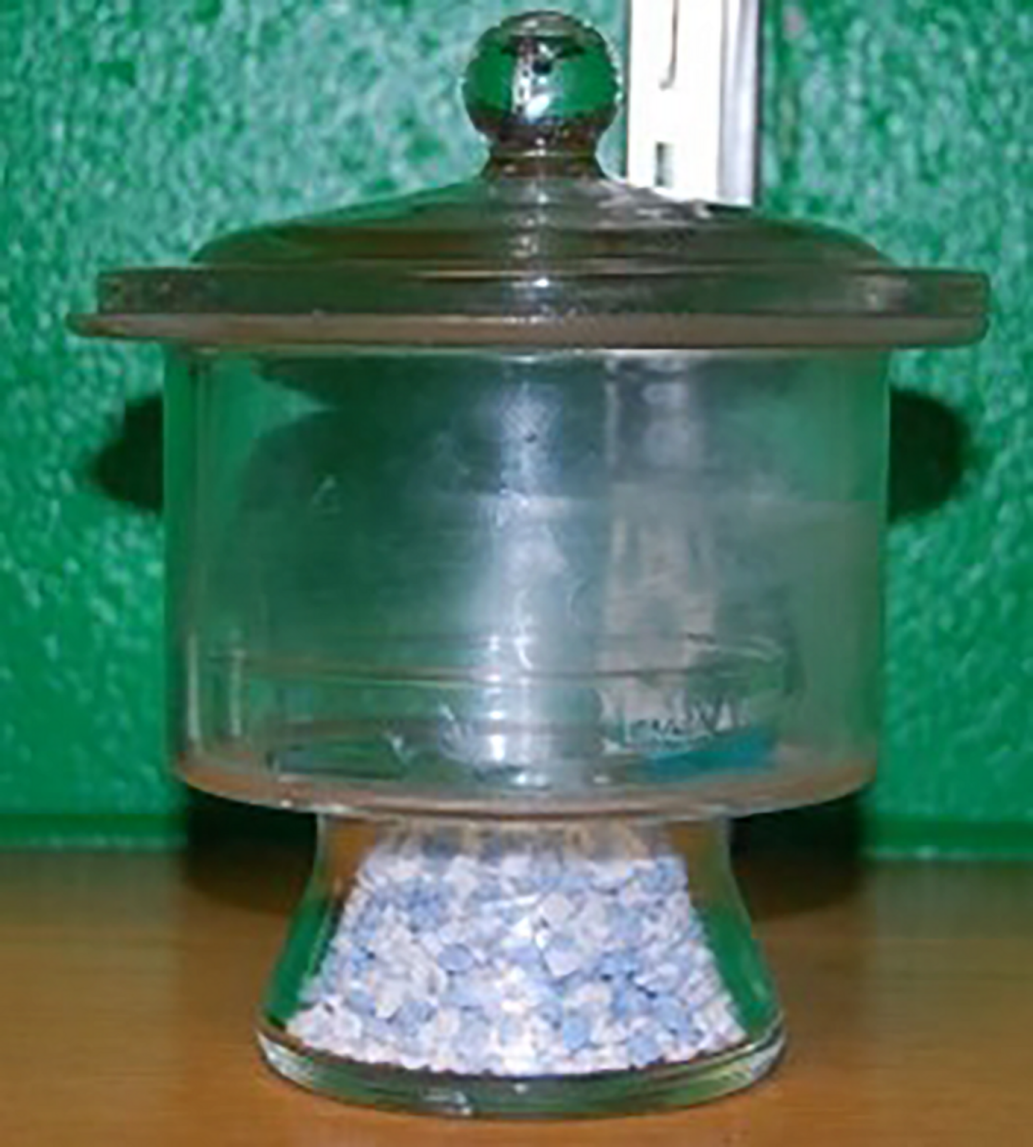
Evaporation inside a desiccator can speed or even control evaporation.

Water evaporation can be complex and slow, but one can speed it up using a desiccator or even a vacuum desiccator if an inert environment is needed (Fig. 5 ▸).

**Figure 6:**
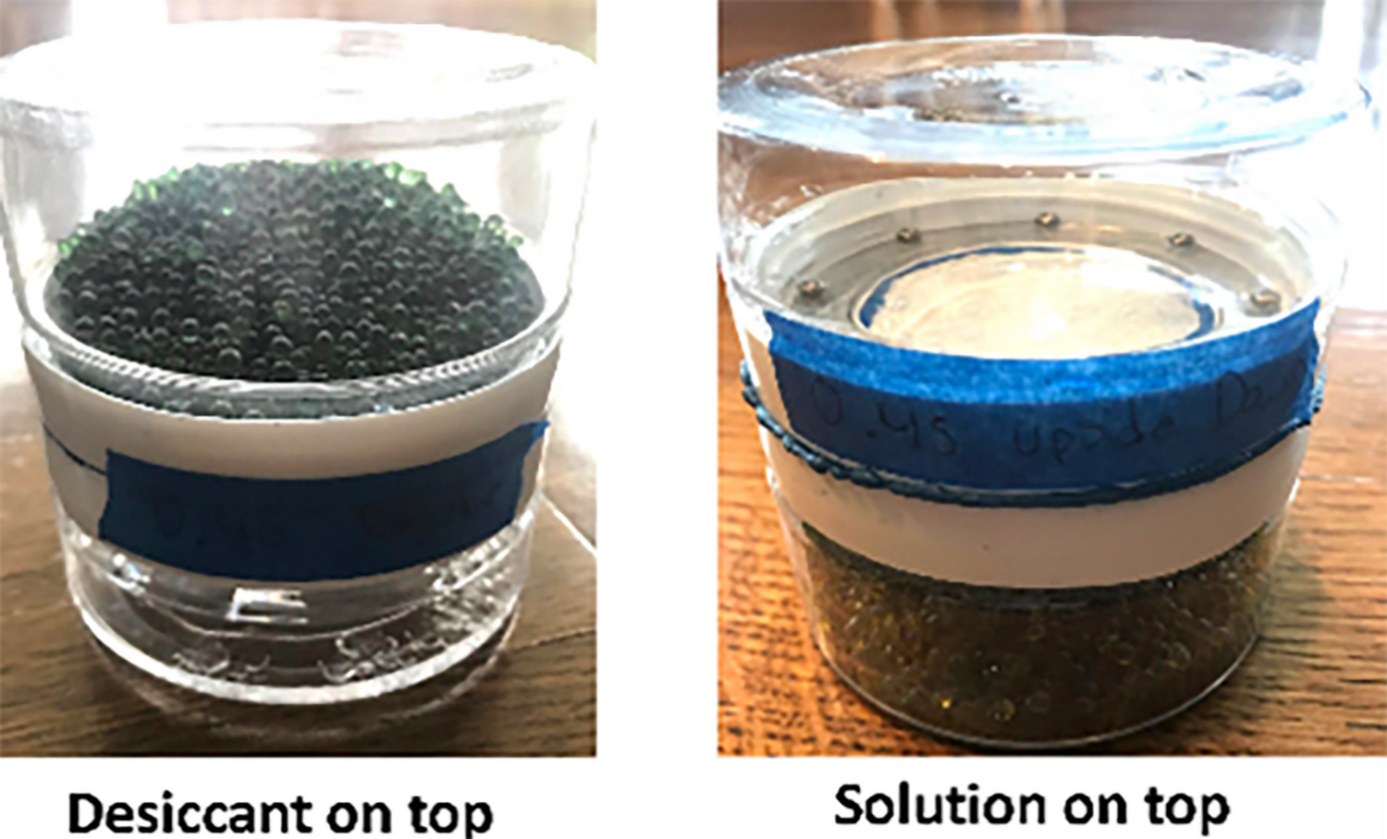
Pictured here are the setups used with the semi-permeable membrane to grow crystal in aqueous solutions (courtesy of Dr Ilia Guzei).

Extensive work crystallizing compounds from water has been done by Ilia Guzei (Guzei *et al.*, 2018 ▸–19) in conjunction with his crystal-growing contest [Wisconsin Crystal Growing Competition (WICGC)], which involves students in the age range 11–18 years old. These experiments have been carried out both on Earth and in space (https://chem.wisc.edu/2019/05/21/2018-19-wisconsin-space-crystal-mission/)! To quote Ilia: ‘We explored the use of a desiccant and a semi-permeable membrane to ensure that an appropriate amount of water is removed from the container with the solution of our compound. The goal was to make the solution supersaturated to induce crystallization. Extensive test of a commercially available desiccant produced consistent results. The experiments on earth and under microgravity conditions produces consistent and similar results.’ The set-up used for the crystal-growing is shown in Fig. 6 ▸.

### Slow cooling

This is the standard recrystallization method that is used very often in inorganic chemistry and air-sensitive work. This method can work very well; remember to follow the rule ‘soluble hot, insoluble cold’. Here we want to have the crystals form very slowly, so a slow reduction of temperature works the best. It is perfectly fine if material is still left in the solution, we want a nicely formed solid and are not interested in a good yield.

This is by far one of the most popular ways that crystals are grown with air sensitive inorganic compounds that are worked on in a glove box. A freezer at 233–243 K is often employed to cool the vials to get crystals.

To generate reduced temperature slowly, isolation of the material from environmental conditions can help, although generally you most often put these crystals in the fridge or freezer. To reduce the time for the vial and solvent system to cool, one can place the crystallization vial into another container. Some people use a Styrofoam box, others a Dewar with foam lid. We find that a jar with cotton (or absorbent material from shipping) in the bottom works well since you can still see if crystals are growing without disturbing them. Sometimes this is hard with cotton and some students use a plastic petri dish as isolation. This works better than glass, which conducts the cold quicker to the vial with the material to crystallize.

Another route to using slow cooling is to make a saturated solution at high temperature, filter away hair and dust particles, then place the solution back in the oil bath. I prefer to warm the oil bath up and dissolve the material at the higher temperature and then shut off the heat and let the oil bath and crystallization vessel cool slowly to room temperature. Since we are cooling the oil and the vessel it is a very slow process, and this can generate some very nice single crystals. **Caution:***The oil will be hot and can burn, and the compound understudy should be known to be stable and not give off toxic fumes when heated.*

### Use of NMR Tube

Often crystals have been obtained by allowing the solvent to evaporate slowly from the NMR tube. The cap fits tight enough to keep dirt out, but allows evaporation of the solvent and crystals form. The inside of the glass is very smooth, which allows for slow nucleation, often resulting in crystals that grow larger and faster than the nucleation. Although there maybe be other reasons for this working extremely well, I present here mainly the fact that it tends to work well and is something that should be tried.

### Sublimation

**Figure 7:**
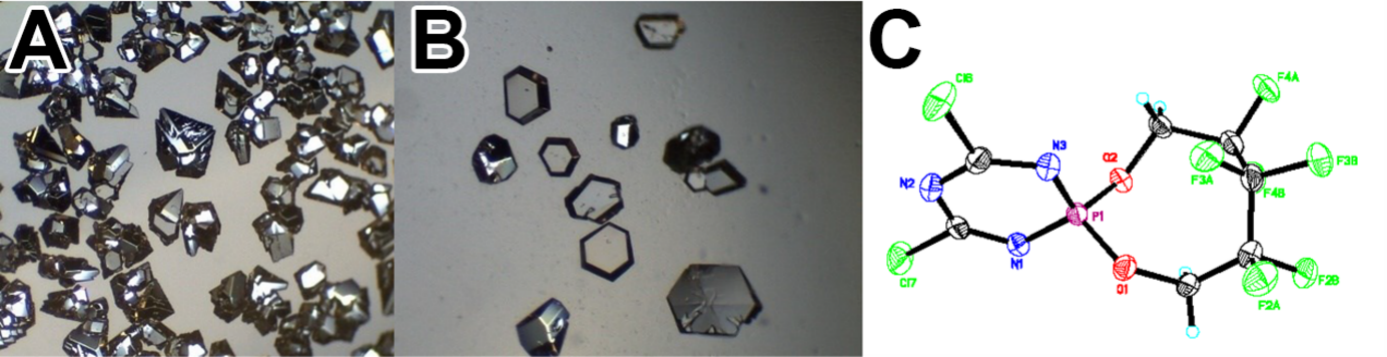
Sublimed crystals, (*a*) too much material (sublimed on top of each other) (*b*) done on mg amounts over a large surface generated very usable crystals with minimum overlap of crystal growth, (*c*) crystal structure from crystals via sublimation (Vij *et al.*, 1997 ▸).

This method often leads to the best quality single crystals. Unfortunately, this cannot be performed for very many compounds and must be performed very slowly and with a small amount of material to get good results. One thing to be careful of is to not have new crystals forming on already formed single crystals (Figs. 7 ▸ and 8 ▸).

### Chiral compounds

Chiral compounds tend to be more difficult to crystallize than racemic compounds because Nature prefers a center of inversion. It can be helpful to prepare derivatives that possess phenyl rings. If absolute configuration is needed, try to incorporate sulfur or heavier atoms. However, this may not be necessary with new modern instruments – check with your local crystallographer.

For crystallization of chiral carb­oxy­lic acids, consider forming the amide with *S*-α-methyl­benzyl­amine. This compound is cheap, typically quite crystalline when incorporated into this chiral group on the compound of interest, and will provide one known stereocenter from which the other stereocenters can be determined.

#### Heavy atoms and crystallization

One thing that can be very helpful in the determination of absolute configuration is to have a heavy atom present such as bromide or iodide (Si, Cl and S also work). For alcohols and amines, consider preparing a derivative using *p*-bromo­benzoate. This usually increases the ability to form good crystals, as well as the determination of the absolute configuration of any stereocenters. It can also be helpful to include aromatic components in the derivative when possible.

### Thermal gradient

Thermal-gradient methods can produce very high-quality crystals. Such methods include slow cooling of sealed, saturated solutions, refluxing of saturated solutions, and gradient (zonal) heating. Gradient heating is used primarily for crystallizing solid solutions or mixtures. Small crystals may sometimes be grown larger by zonally refluxing a supersaturated solution. Larger crystals may be grown either by decreasing the thermal gradient or by cyclic heating and cooling of the sample.

Thermal-gradient heating sometimes works indirectly. If you set up the crystallization apparatus by the cooling vent, one side of the apparatus is cooler than the other and this changes the crystallization properties and can cause crystal formation.

Another way to generate temperature changes, both warm and cold, is the use of a programmable incubator. This allows you to be able to lower the temperature, get a couple of nucleation sites, and then warm slightly to get back into the crystal growth phase of crystallization. Annealing is also possible and sometimes provides good crystals. (Annealing = ramp between cooling and warming cycles.)

### Counter-ions or ionization

Probably the best thing one can do to promote crystallization of an anion or cation is to change the counter-ion. Counter-ions that are generally the same size usually pack well.

The counter-ions most likely to cause difficulties are Et_4_N^+^, Bu_4_N^+^, BF_4_^−^, and PF_6_^−^. Some alternative counter-ions that are usually ordered are trifluoromethanesulfonate, BPh_4_^−^, Me_4_N^+^, (Ph_4_P)_2_N^+^, and Ph_4_As^+^. (Note that salts containing Ph_4_As^+^ are toxic.)

If the compound is neutral and does not crystallize, or if it is a liquid, consider creating an ion. Deprotonation or proton­ation can be performed to generate a salt, which then may crystallize. This is a good way to confirm the identity of the material.

### Co-crystals and molecular framework crystallization

Some have had success with growing compounds in the presence of other compounds, or co-crystallization (Blagden *et al.*, 2008 ▸). This incorporation of another molecule typically occurs when the solvent of crystallization gets trapped in a void in the lattice growth. Pharmaceutical companies have co-crystallized compounds for years (Karagianni *et al.*, 2018 ▸) and there is much in the literature regarding this aspect. Oftentimes, making use of acid--base chemistry or hydrogen bonding can have a marked effect on crystal growth (Sarma *et al.*, 2009 ▸).

The use of tri­phenyl­phosphine oxide (TPPO) has been seen to be a useful co-crystallant for some years in inorganic chemistry and has been reported to be useful for organic molecules that are proton donors (Etter & Baures, 1988 ▸). Alternatively, you can use mechanochemical co-crystallization, which involves grinding material together to generate co-crystals (Friščić & Jones, 2009 ▸).

A final group of co-crystals can be thought of as being formed by incorporating the compound of interest or guest molecule into the small vacant regions in the lattice around large, rigid host molecules, clathrates (Rissanen, 2017 ▸). This is referred to as molecular framework co-crystallization. Two such examples of the use of the outside of clathrate are sponges (Inokuma *et al.*, 2013 ▸) and chaperones (Krupp *et al.*, 2020 ▸).

### Reactant diffusion

This is performed when the compound is very insoluble and difficult to work with after it is formed. Perform the final reaction on a small scale compared to the surface area of the two reactants. Layer one reactant on the top of the other reactant and allow diffusion to control the reaction rate and crystal formation.

The other unique way might be to use a U-tube. If you put a glass frit in the bottom of the U-tube, then put one reactant on one side and the other reactant on the other, the two would diffuse through the frit and possibly form crystals as they react. The type of frit can control the diffusion rate.

Material chemists rely on this type of crystallization a lot, and hope to grow single crystals when preparing their solid material.

### Macro methods

Protein crystallographers use different techniques to grow their crystals. Some people have used these techniques to grow single crystals (Bergfors, 2001 ▸) of small molecules. Current limits on this technique are that the molecule should have a high solubility in water or alcohols or mixture. Advances are being made by companies to help the crystal growing of small molecules using these techniques, even with small amounts of organic solvents in the solution (Hampton Research Catalogue, 2025 ▸).

This technique is to saturate the solution with a smaller cation or anion than your compound (complex), this then will slow crystallization down, but the larger compound comes out of solution leaving the solution saturated with small anions (cations) that you are not interested in. This is essentially how protein chemists grow their crystals, the salts and buffer occupy the solvent, leaving the protein no choice but to fall out of solution, either as a crystal or as another solid form (Drenth, 1994 ▸).

### Seeding the solution

This is a useful method when one of the other methods provides crystals that maybe of reasonable quality, but they are too small to give proper diffraction. Collect some of the crystals, with the mother liquor (it is best if the seed crystals do not dry out), then deposit these (*carefully*) into a fresh or newly created saturated solution.

Seeding a solution with similar crystallized material can also work. Sometimes you have a similar compound that gives good crystals, and you can use one of these crystals as a seed.

### Odd methods

There are many odd methods that have been known to work. Some of these methods have proven to be the only way to get single crystals of the material.

Melting the compound and letting it recrystallize! This can be tried in a melting point tube; once the sample is melted, turn off the heat and leave the tube in place to allow for slow cooling.

Crystal growth in gels for certain materials works very well and has been shown to be successful for many compounds, such as transition-metal oxalates (Chauhan & Arora, 2009 ▸) and proteins. Crystal growth under oil has also been tried on small molecules in our hands, but we have had no success. Some people have reported that they have been successful by crystallizing the compound in the presence of a boiling chip, glass chip and even in some rare cases in the presence of some crystalline mineral powders.

### Combining attempts

In many cases, the compounds are crystallized using a combination of these techniques, more often for organometallic complexes, but it works. Solvent layering combined with putting it in the fridge or the freezer. Temperature range also varies and depends on such factors as: solvent freezing point, available cool places, and the best temperature to get the crystals. In many cases, if freezing at 238 K or lower it is good to not place the vial directly on the metal or plastic shelving. Place a warm paper towel, or better a piece of the absorbent material generally shipped with chemicals.

## Learning from attempts

You can learn from the results of the attempts at crystallization that you have performed. As the chemist, you are in the best position to understand the compound you are trying to grow. What is the compound’s solubility, reactivity and hydrogen-bonding potential? The type of crystal grown can lead to understanding of the possible changes that may be needed to get better crystals.

Macromolecular chemists have been employing this strategy for decades (Hampton Research Catalogue, 2025 ▸).

### Solidified oils

**Figure 9:**
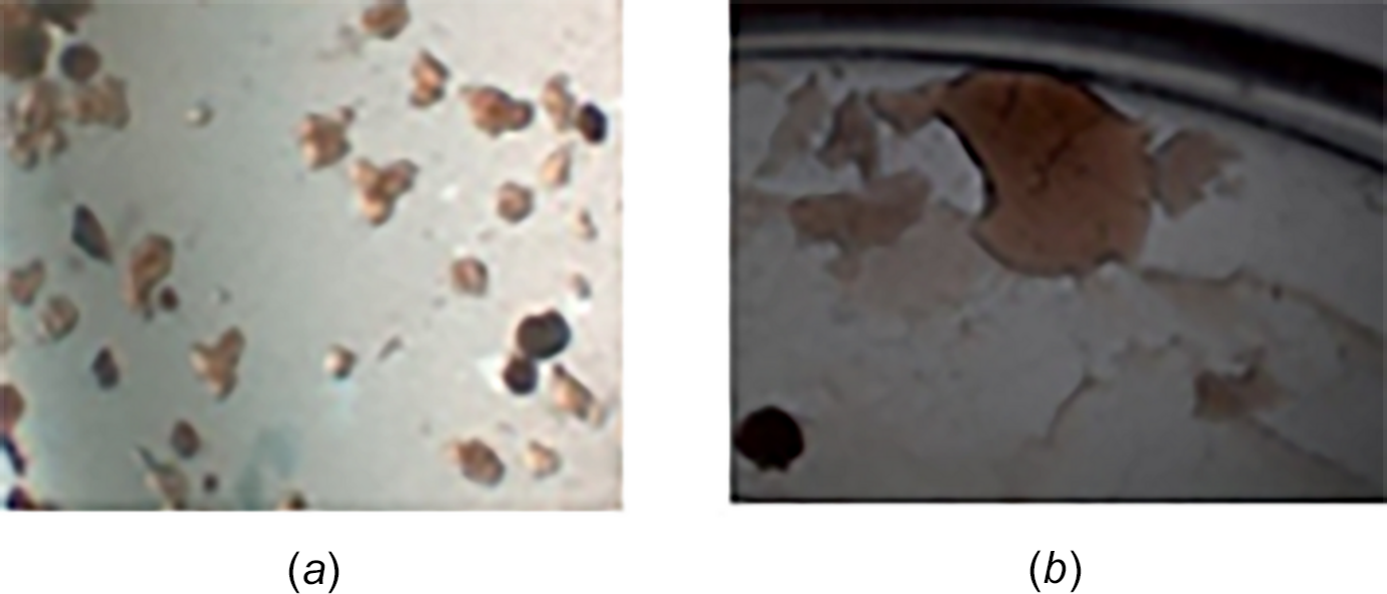
Various solidified oils, (*a*) droplets (*b*) oil pool.

Compounds that generate oils or solids that have a more drop-like shape and are amorphous are generally referred to as a solidified oil (Fig. 9 ▸). In small-molecule crystallography, this generally means that the last solvent seen, or the one that dissolves it into solution, is the one the compound is too soluble in. Remember, that ideally we want the crystallization to start before all the solvent has left or mixed. THF and DMSO have been the worst solvents for this in my experience.

### Dendritic crystals

**Figure 10:**
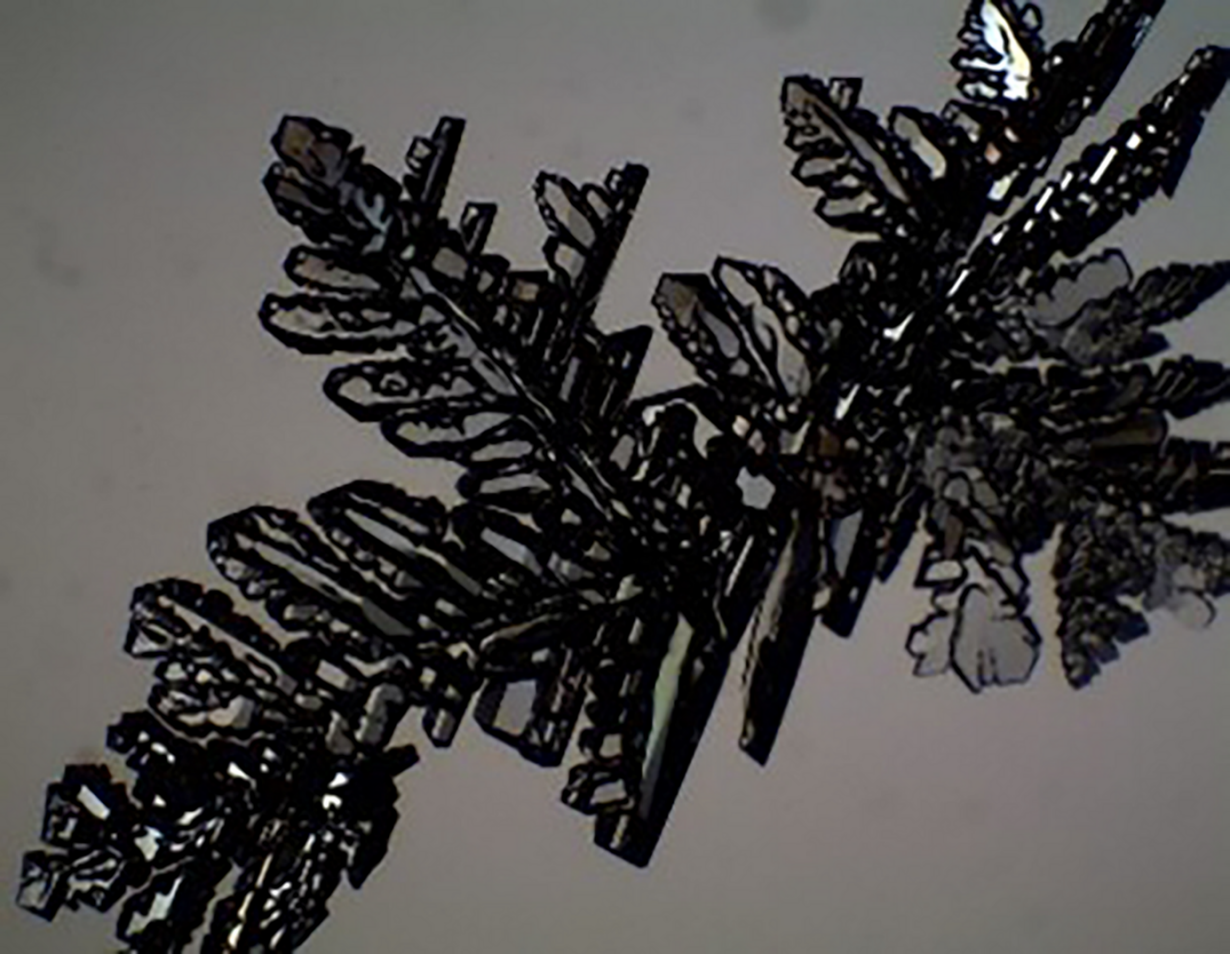
The appearance of a dendritic crystal.

Dendritic crystals appear to have a very tree or snowflake-like appearance (Fig. 10 ▸). Typically, in the early days this would require the growth of the compound in a different solvent to get something large enough, but with today’s instruments sometimes taking a small branch will work.

### Microcrystalline

**Figure 11:**
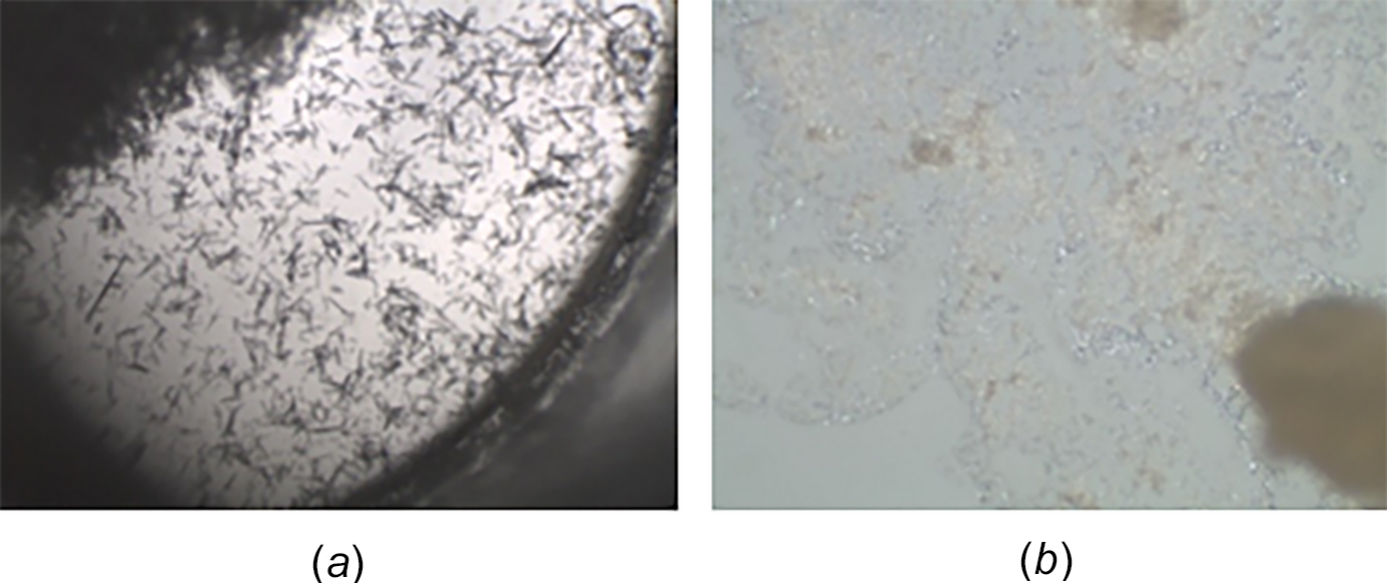
Microcrystalline material

Microcrystals occur when the crystallization occurs too fast, the nucleation process removes the bulk of the material before the crystal growth phase is reached (Fig. 11 ▸). Although these may not work, newer instruments can do smaller crystals, this is a good sign that crystals can be grown. Create a situation where the crystals grow slower, *i.e.* less concentration, slower cooling if cooling. It is this author’s opinion that with some tweaking of the crystal growth, one can get large enough crystals from these types of results.

### Powder

**Figure 12:**
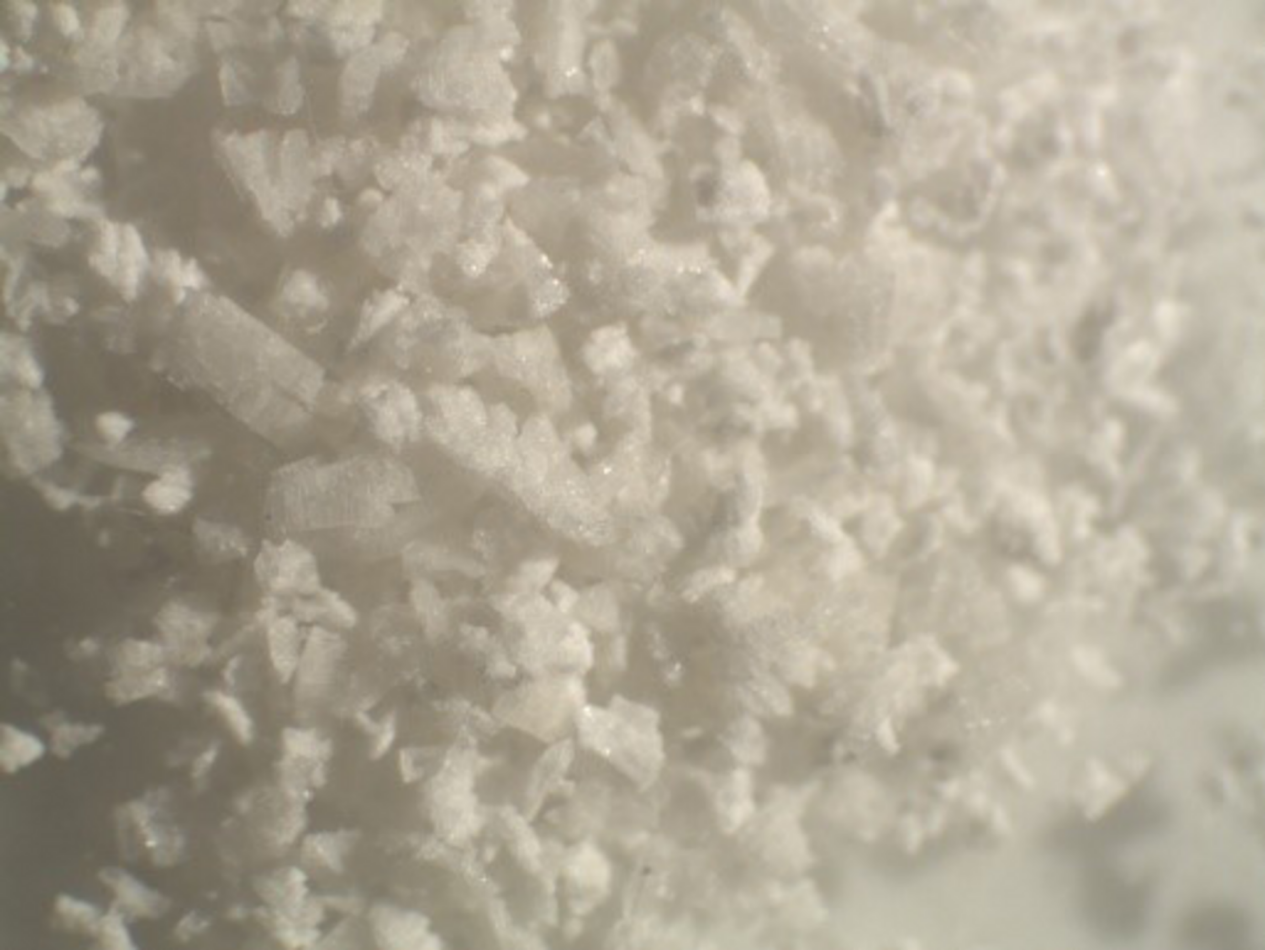
Powder formation generally implies the material came out of solution too fast.

This often also means that the material has come out of solution too fast and the process of precipitation or crystallization needs to be slowed down (Fig. 12 ▸). However, this is not a guarantee that a crystal can be grown. Sometimes solvent changes and the way you grow the crystal needs to be modified to get a crystal. There have been times when I could not grow a crystal of a material that powders out. I always believe that means I have not yet stumbled on the best method to generate a crystal.

### Cracked crystals

**Figure 13:**
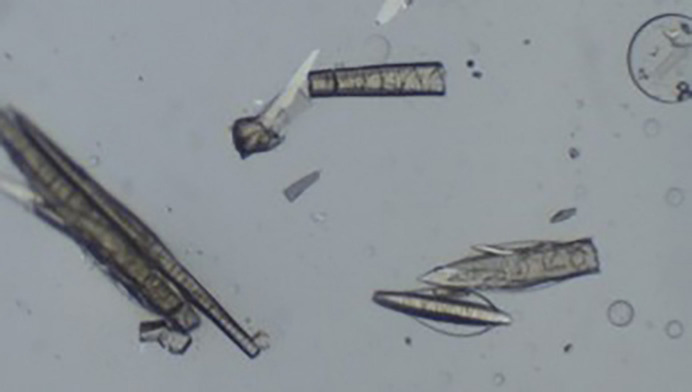
Appearance of cracks in the crystal can mean that there is solvent loss.

Cracked crystals can occur from slow evaporation and generally means that the solvent was trapped in the crystal and then evaporated, which cracked the crystal (Fig. 13 ▸). This can also be seen when working with the crystals to mount them on the instrument. These types of crystals are common when di­ethyl ether or THF is one of the solvents. My suggestion is to substitute pentane for di­ethyl ether. THF is sometime hard to replace, so this situation may require some special handling (cold). If evaporating the solvent, do not allow it to go to dryness. If the cracks are generated when the crystals are removed from the solvent, the crystallographer or you will need to employ some of the mounting techniques that can avoid this issue.

## Vary the vial or glassware

Flat, or round, or mass spectrum vials have been known to provide different results. We have seen crystals grown in almost any and every type to glassware. The idea here is that the compound may need a different type of surface to nucleate and thereby generating a different, more useful crystal form.

The use of siliconized glassware as the crystallization vessel can help when a large number of micro-crystals is formed. This can reduce the number of nucleation sites, as well as help when the crystals received like to adhere to the glass walls of the container and therefore do not allow for the collection of crystals. Sometimes this will reduce the number of nucleation sites. I have personally had success with these types of vials (Siliconized Glassware, 1994 ▸).

## Conclusion: key factors to good crystals

There are some main points that I would like to highlight here to be sure you consider them as you embark upon the journey to grow crystals. I also highlight some of the limitations to X-ray crystallography that one always needs to be aware of when interpreting and reviewing crystal structures.

**·** Solvent – choosing the right solvent or solvent system is very important.

**·** Nucleation – generating only enough nucleation sites that you get a few large crystals and not lots of small ones.

**·** Mechanics – the physical method that takes place to get the crystal, diffusion, evaporation, gas-solid change. The location of the apparatus that is growing the crystal.

**·** Time – the longer it takes to grow the crystals, generally the better. Unfortunately, this does not always apply.

**·** Patience, patience.

## Limitations to crystallography

**· Requires single crystals**. This by far is the greatest limitation to X-ray diffraction analysis. Sometimes one can achieve a solution with complex single crystals, but they must diffract in the X-ray beam. ***No crystal, no information.***

**·** **Crystal quality governs the quality of results obtained.**

**· Only one crystal of the bulk material. Remember that we are looking at one small crystal in the entire bulk of the material. **Might have chosen an impurity.

**· Chirality determination is dependent on how good the crystal and data, as well as contents of the asymmetric cell. Easily determined if one chiral center is known.** Might have chosen an impurity.

**Modern instruments can sometimes generate data sufficient to determine chirality with only H, C, N and O atoms present.**

## Supplementary Material

Poster for NASA reference. DOI: 10.1107/S2056989025007807/oi2022sup2.pdf

## Figures and Tables

**Figure 4:**
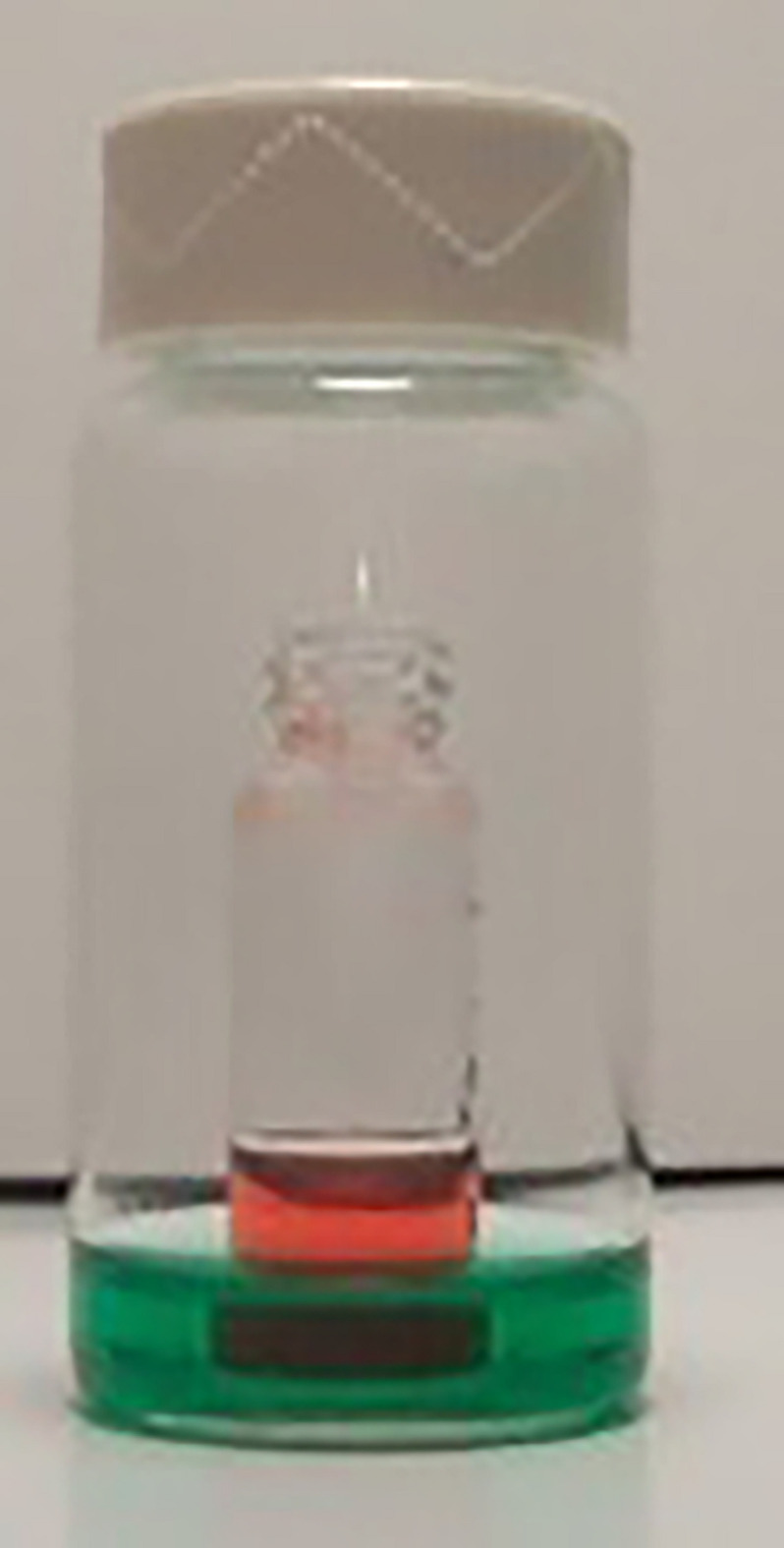
A typical vapor diffusion set-up: in this scenario we expect that the green (outer) solution will move to the red (inner) solution containing the compound and cause crystallization.

**Figure 8:**
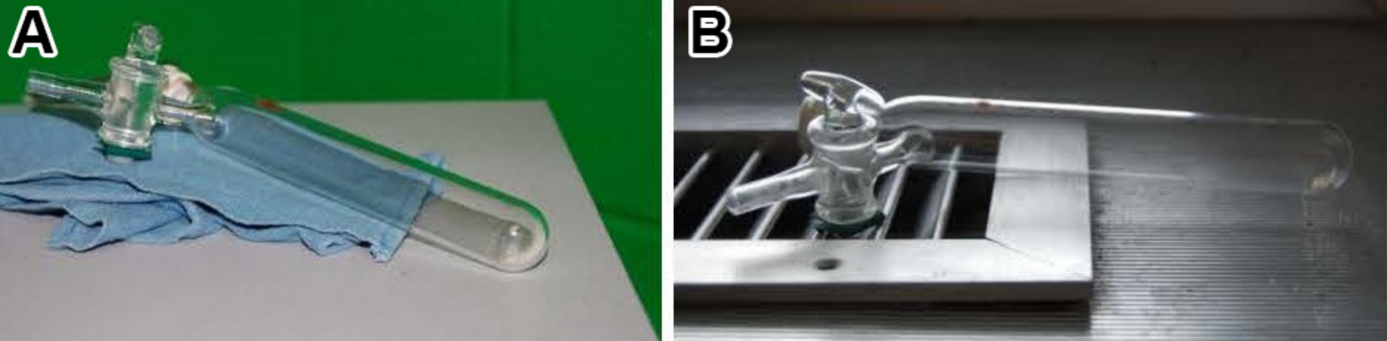
Simple sublimation can occur using a Schlenk flask under vacuum and then generating an (*a*) heat (top of an oven) or (*b*) cooling gradient (across a cooling vent).

**Table 1:** Typical solvents used for growing single crystals of organic compounds

Water – alanine, organic acids	Ethanol (CH_3_CH_2_OH) – soluble hot, insoluble cold
Methanol (CH_3_OH) – soluble hot, insoluble cold	Iso­propanol
Di­chloro­methane^*a*^ (CH_2_Cl_2_) {DCM}	Dioxane
Aceto­nitrile (CH_3_CN)	Iso­propyl acetate (CH_3_C(O)O(C(CH_3_)_2_)
Toluene (C_6_H_5_CH_3_)	1,2-Dichoro­ethane (ClCH_2_CH_2_Cl)
Ethyl acetate (CH_3_CO_2_C_2_H_5_)	Acetone (CH_3_(CO)CH_3_)
Benzene^*a*^ (C_6_H_6_)	Tetra­hydro­furan, THF (C_4_H_8_O)
Methyl­formate	Hexane(s)
Heptane	Pentane
Di­ethyl ether	Petroleum ether
Cyclo­hexane	Cyclo­pentane


**Rare solvents** ^*b*^	
1,1,1-Tri­chloro­ethane (instead of DCM^!^)	Methyl­cyclo­hexane (instead of cyclo­pentane)
Meth­oxy­benzene	Pyridine
Dilute HCl	Phosphate buffered pH
Acetic acid, tri­fluoro­acetic acid	Formic acid
	

Notes: (*a*) These are solvents that are now or have been limited use, or are considered controlled substances. (*b*) *N*,*N*-Di­methyl­formamide (DMF) and di­methyl­sulfoxide (DMSO) can be used as a ‘last resort’ when the compound is insoluble in all other solvents. The challenge with these solvents is that often times compounds are *so* soluble in them that they do not crystallize back out. Layering or vapor diffusion works best in these cases. Water is an option that has proven useful for peptides, and can be combined with alcohols, aceto­nitrile or DMSO as co-solvents. Lastly, a list of 107 solvents is provided in Spingler *et al.* (2012 ▸).

**Table 2:** Potential solvent choices for vapor diffusion

Solvent	Anti-solvent
Water	Dioxane
Methyl­ene chloride^*a*^	Di­ethyl ether
Methyl­ene chloride^*a*^	Cyclo­pentane
Methyl­ene chloride^*a*^	Hexanes^*b*^
Aceto­nitrile	Pentane, di­ethyl ether or diiso­propyl ether
Ethyl acetate	Hexane, pentane, di­ethyl ether or diiso­propyl ether
Iso­propyl acetate	Di­ethyl ether or diiso­propyl ether
Ethanol	Cyclo­hexane
Methanol	Hexane
Methyl­formate	Cyclo­pentane
Methyl­formate^*c*^	Hexanes^*b*^

Notes: (*a*) This is considered a controlled substance. (*b*) If a paper says hexanes were diffused into di­chloro­methane, in reality this was a controlled evaporation. (*c*) Methyl­formate is being used as a substitute for di­chloro­methane.

**Table 3:** Table of vapor pressures at room temperature.^*a*^ The solvent with the lower vapor pressure will diffuse into the solvent with the higher vapor pressure

Solvent	Vapor pressure (torr)
Water	21.0
Di­ethyl ether	34.6
Pentane	36.1
Di­chloro­methane^*b*^	40.7
Acetone	56.5
Chloro­form	61.3
Methanol	64.1
Hexane	68.7
Ethyl acetate	77.1
Ethanol	78.4
Benzene^*b*^	80.1
Aceto­nitrile	81.8
Heptane	98.4
Toluene	110
Octane	125

Notes: (*a*) Vapor pressure data taken from various sources and used only as a guide for vapor diffusion experiments. (*b*) These are solvents that are now or have been limited in use, or are considered controlled substances.
