# Supplementary material for: Getting crystals your crystallographer will treasure: a beginner’s guide
Source: Acta Crystallogr E Crystallogr Commun. 2025 Sep 9;81(Pt 10):895–905. doi: 10.1107/S2056989025007807 (PMC12498048; doi:10.1107/S2056989025007807)
Supplement: Supplementary file 1 [file e-81-00895-sup2.pdf]

SUPPLEMETAL MATERIAL FOR:

Getting Crystals Your Crystallographer Will Treasure: A Beginners Guide

Authors

Richard J. Staples[a]*

[a]Chemistry, Michigan State University, 578 S. Shaw Lane, East Lansing, MI, 48824, USA

Correspondence email: staples@chemistry.msu.edu

Crystallization of KDP in Microgravity With a Selectively Permeable Membrane.

Joseph Quinn[1], Kaitlyn Twesme[2]

[1] Home School. Whitefish Bay, WI, [2] Lake Mills High School. Lake Mills, WI

Working Concepts Of The Experiment

Introduction

The project is to grow Potassium Dihydrogen Phosphate (KDP) crystals efficiently in microgravity using the evaporation method. The experiment is being done in microgravity to eliminate the effects of convection. The hypothesis is that the crystallization will be faster and form higher quality crystals in microgravity relative to ground conditions. To test this hypothesis, we have designed a special device that uses two chambers separated by selectively permeable membrane to allow for evaporation in microgravity. Results will be compared between experiments on Earth and aboard the International Space Station.

Background

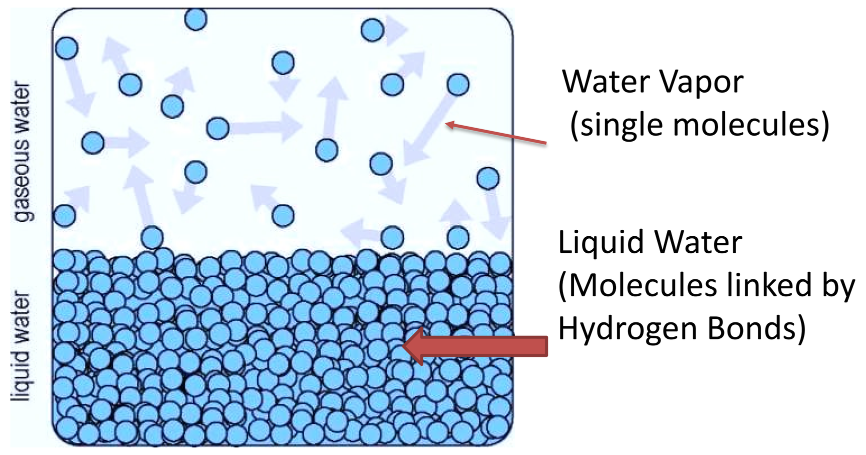

Evaporation of water. Liquid Water molecules are held together by hydrogen bonds. The hydrogen bonding creates a large structure that can not fit through the small holes of the membrane. After evaporation, water molecules exist as isolated molecules that can pass through small holes.

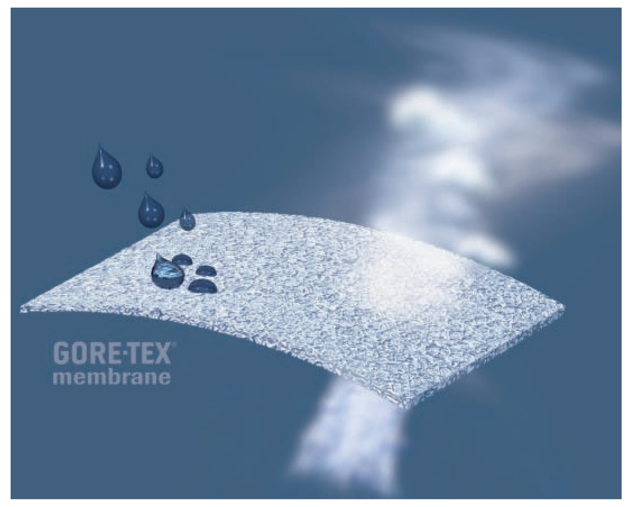

Water vapor passes through Gore-Tex

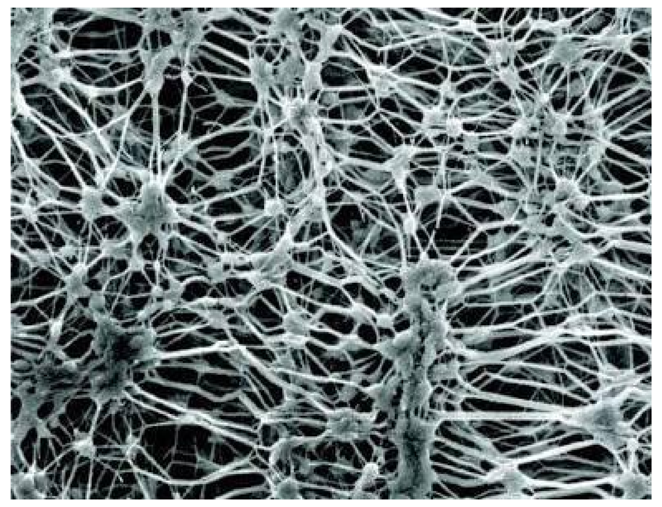

Electron micrograph of a PTFE membrane

PTFE is filled with billions of tiny pores, each roughly 700 times bigger than a water vapor molecule. The pores allow water vapor to easily pass through, but are 20,000 times smaller than a water droplet, causing it to be impenetrable to liquid water. Gore-Tex is a special type of PTFE that is laminated to a base layer for extra strength. We have tested both Gore-Tex and a generic PTFE membrane.

Experiment Hardware and Tests

Membrane Testing

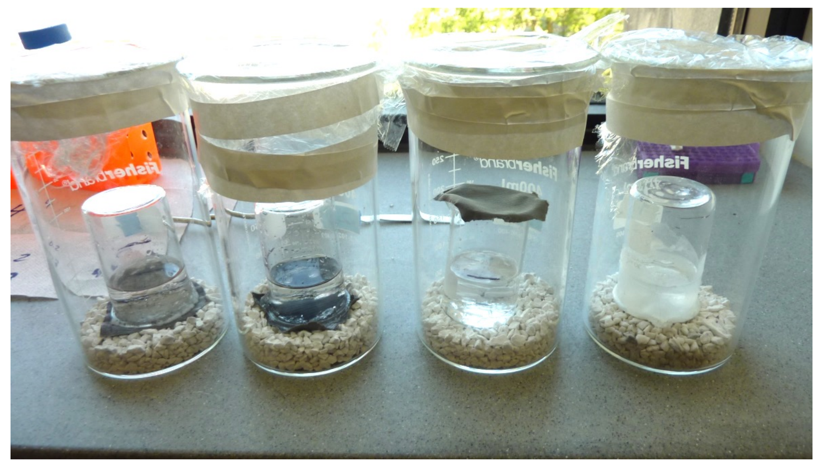

Testing Gore-Tex and PTFE membranes. The first tests were done with Drierite desiccant and meant to test desiccant efficiency, membrane permeability, and membrane adhesion. Later on, we used silica desiccant for testing

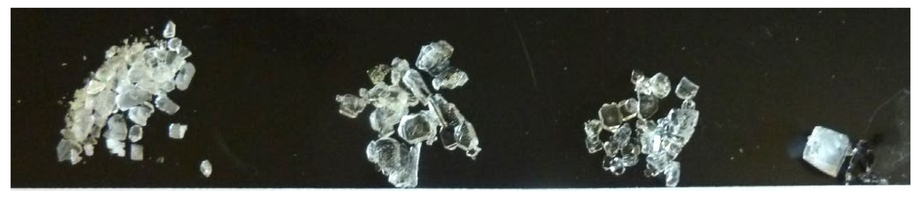

Gore-Tex Upside down Crystals on Gray side*	Gore-Tex Upside down Crystals on Black side	Gore-Tex Membrane Facing up	PTFE Membrane Facing down

Crystals obtained from the membrane tests. After running the experiment for 14 days, the crystals were rinsed with water, weighed and photographed. Both generic PTFE and Gore-Tex produced crystals. However, crystals stuck tightly to the generic PTFE and had to be scraped off.

Design of closed system for crystallization

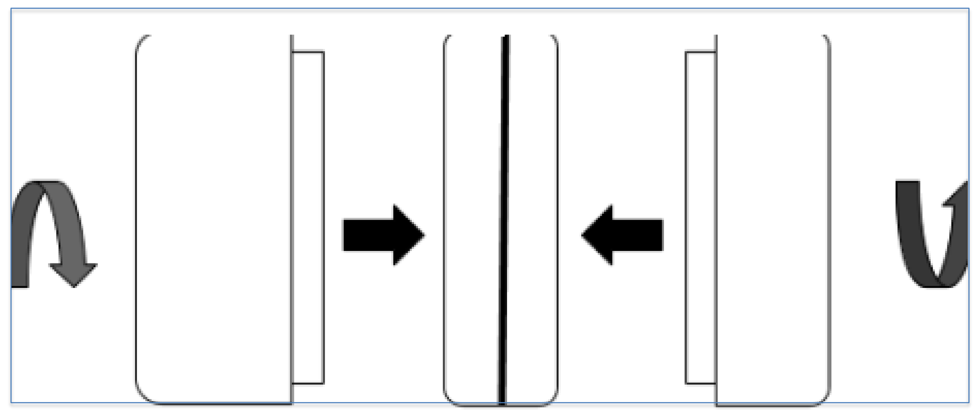

Desiccant jar — Selective membrane assembly. — Crystal solution jar

Design of the crystal growing assembly. The experiment hardware is designed to separate the desiccant from the solution and keep the experiment in a closed system. It is made of two screw lid containers, an 8oz jar, and a 4oz jar held together by a selective membrane assembly to connect both the jars. The assembly is a sandwich comprised of a PTFE or Gore-Tex layered membrane, RTV, and two screw lids from the jars which are all held together by two plastic rings on either side of the assembly screwed together with metal screws.

Color-Indicating Desiccant

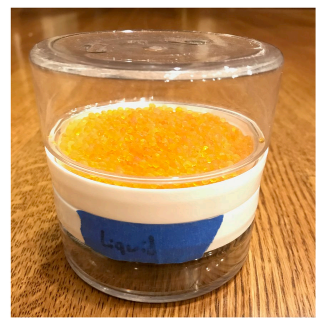

Desiccant is yellow before the experiment

To keep the experiments in a closed system, a desiccant is required to absorb evaporated solution, and allow more to be evaporated. Silica desiccant was chosen because of its low density, and higher efficiency. The chosen type of silica bead also was non-toxic and had a color changing indicator

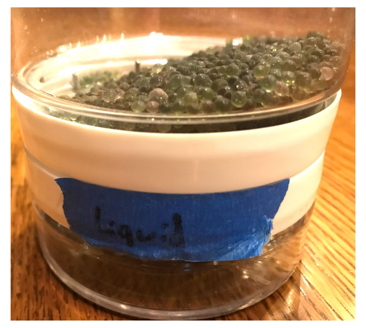

Desiccant is green after the experiment

Results

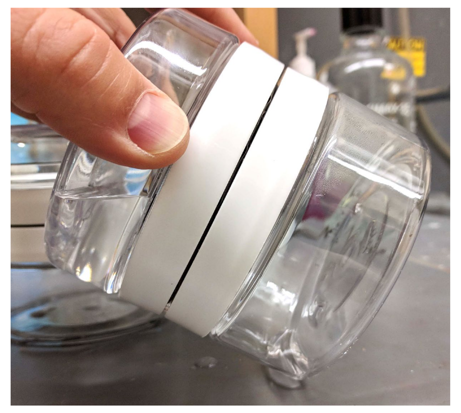

Leak testing. Early testing revealed that the hardware was leaky. This problem was resolved by using RTV sealant instead of rubber O-rings.

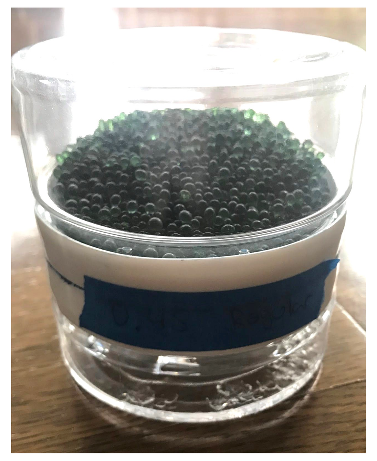

Desiccant on top

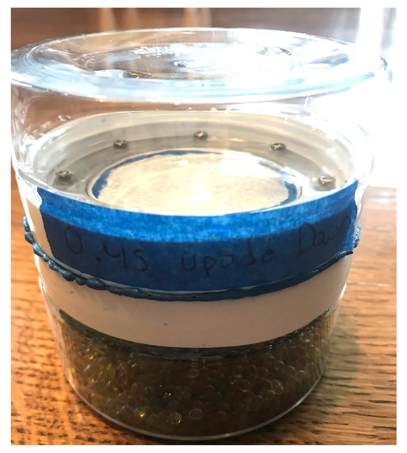

Solution on top

The crystallization chamber was tested in two orientations. In microgravity conditions, we expect both the solution and desiccant to contact the membrane. Therefore, to simulate microgravity conditions, we tested the chamber in two orientations, Desiccant on membrane and Solution on membrane. Crystallization occurred in both orientations.

	Average crystal weight desiccant on top	Average crystal weight solution on top
.44 PTFE	5.4 g	2.0 g *
.22 PTFE	4.2 g	5.0 g
Gore Tex	4.1 g	5.0 g

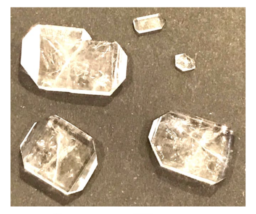

Gore Tex desiccant on top.

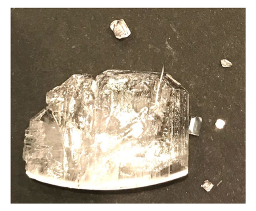

0.45 PTFE desiccant on top.

Results of membrane assembly tests. Crystals were grown in two different orientations for each of the two membranes. After 14 days of growth the crystals were isolated and weighed. Examples of crystals grown under the membrane are shown at the left for Gore-Tex and generic 0.45 PTFE. *The crystals growing on top of the generic PTFE stuck very tightly and had to be removed by scraping.

Conclusions

Ground testing has shown that a selectively permeable PTFE membrane can be used to allow crystallization in a closed system. Both Gore-Tex and a generic PTFE membrane work, but crystals may stick too tightly to the generic PTFE under ground conditions.

Future Plans

The performance of Gore-Tex and generic PTFE in microgravity is not yet known and will be investigated aboard the International Space Station. The experiment will travel to the Space Station on a Falcon 9 rocket (SpaceX CRS-17). Astronauts will assemble the device and initiate the experiment in microgravity. Crystals grown on the Space station will be analyzed for size, shape and quality and compared to crystals grown on Earth.

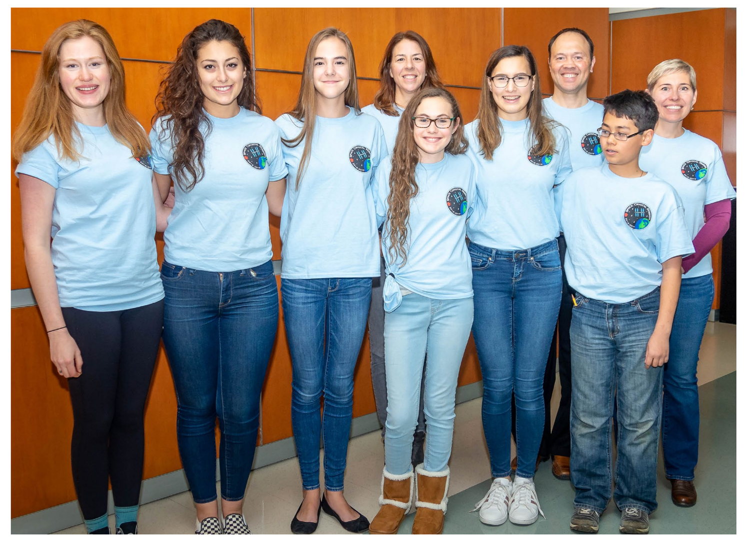

Acknowledgements

2019 WISCM team members: Kristin Kiley, Joana Pashaj, Payton Kelly-VanDomelen, Kalea Greenfield

Mentors: Dr. Ilia Guzei and Dr. Galina Bikzhanova, Stephanie Twesme.

CASIS Staff: April Spinale

DEPARTMENT OF Chemistry UNIVERSITY OF WISCONSIN–MADISON

Wisconsin Crystal Growing Contest 2018

SPACE CRYSTAL MISSION 2018-2019

SPACE STATION EXPLORERS Powered by CASIS
